# Intermittent preventive treatment for malaria among children in a refugee camp in Northern Uganda: lessons learned

**DOI:** 10.1186/s12936-017-1869-x

**Published:** 2017-05-23

**Authors:** Matthew E. Coldiron, Estrella Lasry, Malika Bouhenia, Debashish Das, Peter Okui, Dan Nyehangane, Juliet Mwanga, Celine Langendorf, Greg Elder, Léon Salumu, Rebecca F. Grais

**Affiliations:** 1 Epicentre, 8 Rue Saint-Sabin, Paris, France; 2 Médecins Sans Frontières, New York, USA; 3 National Malaria Control Programme, Ministry of Health, Kampala, Uganda; 4 Epicentre, Mbarara, Uganda

**Keywords:** Malaria, Anti-malarials, Artemisinin, Quinolines, Refugee, South Sudan, Uganda

## Abstract

Northern Uganda hosts a large population of refugees from South Sudan, and malaria is one of the major health problems in the area. In 2015, intermittent preventive treatment for malaria (IPTc) was implemented in two refugee camps among children aged 6 months to 14 years. Three distributions of dihydroartemisinin–piperaquine (DP) were conducted at 8-week intervals. The first dose was directly administered at IPTc distribution sites and the second and third doses were given to caregivers to administer at home. A multi-faceted evaluation was implemented, including coverage surveys, malaria prevalence surveys, reinforced surveillance, and pharmacovigilance. Programme coverage exceeded 90% during all three distributions with a total of 40,611 participants. Compared to same period during the previous year (only available data), the incidence of malaria in the target populations was reduced (IRR 0.73, 95% CI 0.69–0.77 among children under 5 years old; IRR 0.70, 95% CI 0.67–0.72 among children aged 5–14 years). Among those not targeted for intervention, the incidence between the 2 years increased (IRR 1.49, 95% CI 1.42–1.56). Cross-sectional surveys showed a prevalence of parasitaemia (microscopy or PCR) of 12.9–16.4% (95% CI 12.6–19.3) during the intervention, with the highest prevalence among children aged 5–14 years, but with a large increase 8 weeks after the final distribution. A total of 57 adverse events were reported during the intervention period, including one severe adverse event (death from varicella). Adverse events were of mild to moderate severity, and were mainly dermatologic and gastrointestinal. This is the first documentation of an IPTc programme in a refugee camp. The positive impact of DP on the incidence of malaria, together with its favourable safety profile, should lead to further use of IPTc in similar settings. Expanding coverage groups and decreasing intervals between distributions might provide more benefit, but would need to be balanced with the operational implications of a broader, more frequent distribution schedule.

## Background

Clear recommendation and guidelines exist for several preventive treatment strategies for malaria, including seasonal malaria chemoprevention (SMC) with sulfadoxine–pyrimethamine and intermittent preventive treatment of infants (IPTi) and pregnant women (IPTp) [1–3]. Such clear guidance does not exist for older children, although many different variations of intermittent preventive treatment of children (IPTc) programs have been implemented. Dihydroartemisinin–piperaquine (DP) has been shown to be effective when used for IPTc at monthly intervals, while there was little effect if the interval between distributions was 3 months [4]. Nonetheless, this trial, like many others, was performed in schoolchildren in an open setting. There is scant evidence on the effects of such programmes in the setting of a refugee camp, a closed environment with a vulnerable population. On the other hand, there is strong evidence of the burden of malaria in refugees in endemic areas [5], which may be higher than the burden of disease among their host populations [6]. While concerns have existed about repeated dosing of dihydroartemisinin–piperaquine (DP), large studies have not shown problems with its safety [4, 7].

In March 2015, over 630,000 South Sudanese refugees were living in Uganda [8]. Following a large number of arrivals in the first half of 2014, the medical humanitarian organization *Médecins Sans Frontières* was present in several camps in the Adjumani District of northern Uganda between January 2014 and July 2015, providing both curative and preventive care to over 40,000 persons. Over 38,000 cases of malaria were notified in 2014 in two camps located in Ayilo (Ayilo 1 and Ayilo 2), Adjumani District, despite distribution of long-lasting insecticide treated bed nets (LLINs) upon arrival into camps. Like much of Uganda, the Adjumani District is situated in an area of high, stable transmission of malaria, and the predominant species is *Plasmodium falciparum*, responsible for 90–98% of malaria episodes. In the West Nile region of Uganda (which includes Adjumani District), the prevalence of malaria among children aged 0–59 months was 51.3% by rapid diagnostic test (RDT) and 27.5% by microscopy in the 2014–2015 malaria indicator survey [9].

An expanded IPTc programme was put in place in the two Ayilo camps between March and July 2015. The programme consisted of administering a 3-day course of DP to all children aged between 6 months and 15 years every 8 weeks. A total of three rounds were given (March, May, July). DP was chosen for this programme because of its long protective period, its registration in Uganda, and its status as a second-choice artemisinin combination therapy (ACT) (artemether–lumefantrine is the first-choice ACT). The 8-week interval was chosen as a compromise; while several studies have shown superiority of monthly IPTc distribution [4], this was not deemed logistically feasible in this context. The distributions were organized every 8 weeks at 11 fixed sites; distributions lasted 2 days, with 1 day of catch-up. Children were judged for their eligibility to receive DP: in addition to allergy to DP, exclusion criteria focused on avoiding drug–drug interactions as described by the manufacturer. In order to simplify evaluation of children, children currently taking any anticonvulsant, antituberculosis or antiretroviral medications were excluded, as were any children who had taken artemether–lumefantrine in the 2 previous weeks. DP (Eurartesim^®^, Sigma-Tau, Italy) was dosed by weight, and the first dose was directly observed at the treatment site. The remaining two doses were given to the child’s caretaker in the original blisterpack, with instructions on how to administer the doses at home each of the next 2 days. For children unable to swallow pills, the pill was crushed and mixed with water and sugar.

The programme in Ayilo was the first large-scale implementation of an IPTc programme in a refugee camp in Africa. Given its scale and its overall context, this study aimed to document this intervention, including its effects on malaria incidence and prevalence, as well as side effects potentially related to repeated doses of DP.

## Methods

The study population consisted of the entire refugee population of Ayilo 1 (area: 3.8 sq km) and Ayilo 2 (area: 2.4 sq km) camps, a total population of 29,855 refugees as well as the members of their host community, population approximately 1000 persons). Approximately 90% of refugees were Dinka and about 10% were Madi. The camps were divided into blocks, each with a population between 2000 and 4000, and the two ethnic groups lived in separate blocks.

Facility-based malaria surveillance was reinforced with additional detail regarding patient age captured. A case of malaria was considered to be fever with a positive RDT [SD Bioline Malaria Ag P.f (HRP-2)]. Because the camps were new settlements, prior historical data were not available to compare incident malaria cases in a time series. The overall number of incident malaria cases for the period March–August 2015 (corresponding to the likely protective period of DP) was compared to the same period of 2014, during which time the overall population of the camps was stable.

A total of four malaria prevalence surveys were carried out: the first three were performed in the week prior to each of the three IPTc distributions, and the fourth was performed 8 weeks after the final IPTc distribution. The population was stratified into three groups (6–59 months, 5–14, 15 years and older). Systematic sampling was used to include 275 households; one person from each age group was randomly selected for participation. After capillary fingerstick, thick and thin smears were prepared and a set of dried blood spots was collected. Point-of-care haemoglobin testing was performed (HemoCue Hb 301, Meaux, France) among children aged 6–59 months. In a research laboratory in Uganda, microscopy was performed on all slides following WHO guidelines [10] in a WHO Centre of Excellence whose microscopists undergo routine proficiency testing. For slides negative by microscopy, qualitative PCR with speciation (Type-it HRM PCR kit, Qiagen) was performed, then quantitative PCR (artus Malaria RG PCR Kit, Qiagen) in turn performed on PCR-positive samples (detection limit 1 parasite/µl).

Community-based programme coverage surveys 2 days after each distribution used systematic sampling, and were stratified by age group (6–59 months and 5–14 years). A total of 250 households were targeted in each survey. Parents of non-participating children were asked why they did not participate in the distribution, and also to describe adherence to the second and third doses of DP at home.

Because of the logistic difficulty of weighing children in a mass treatment context (long lines, fear of scales, hot and dusty conditions), and because of the desire to more easily distribute DP in similar settings, an attempt was made to create a height-based dosing scale. During the first distribution, weight and height data were recorded for all children attending at two distribution sites. A posteriori, a logarithmic curve was fit to these data, and the dose of DP actually administered based on weight was compared to the dose of DP that would have been administered if the dosing had been based on the child’s height.

An existing community-based mortality reporting system was reinforced to ensure that all deaths and serious illnesses in the community were investigated. Programme participants were instructed to return to the health centre for any illness in the days after taking DP. Health centre staff received training about side-effects associated with DP and a dedicated register was put into place to capture adverse events (AE). The causal relationship between DP and the reported AE was judged by the treating physician as related or unrelated [11–13] based on the clinical presentation and the time between administration of DP and the onset of symptoms.

Participation in the IPTc distributions was voluntary. Participants in the malaria prevalence surveys provided written informed consent prior to sample collection. The study protocol was reviewed and approved by the Research Ethics Committee of the Mbarara University of Science and Technology, the Uganda National Council for Science and Technology and the *Comité de Protection des Personnes*, Ile-de-France XI of Saint-Germain-en-Laye, France.

## Results

A total of 40,611 children received DP during the programme between 21 March and 13 July, 2015, an average of 13,537 per distribution.

### Malaria incidence

Among children <5 years, the 6-month incidence was 0.71 cases/person in 2014 and 0.52 cases/person in 2015 (IRR 0.73, 95% CI 0.69–0.77). Among children aged 5–14 years, the 6-month incidence was 0.96 cases/person in 2014 and 0.67 in 2015 (IRR 0.70, 95% CI 0.67–0.72). Among persons aged 15 years or older (who did not receive DP in 2015), the 6-month incidence was 0.37 cases/person in 2014 and 0.55 cases/person in 2015 (IRR 1.49, 95% CI 1.42–1.56).

### Programme coverage and adherence

When considering both card-confirmed and verbally confirmed receipt of IPTc, programme coverage exceeded 90% during each of the three distributions (Table 1). No significant differences were seen by gender or by age.

**Table 1:** Estimated proportion of children receiving a distribution of dihydroartemisinin-piperaquine, Ayilo camps, Uganda 2015

	Distribution 1 March 2015 (N = 1076)		Distribution 2 May 2015 (N = 1249)		Distribution 3 July 2015 (N = 1189)	
	%	95% CI	%	95% CI	%	95% CI
Card-confirmed receipt	87.7	84.3–90.5	83.6	79.3–87.1	78.6	73.8–82.8
Card- or verbally confirmed receipt	93.7	91.3–95.5	95.1	92.4–96.9	95.3	93.0–96.9
Gender						
Male	93.6	90.8–95.6	95.7	93.9–97.0	94.3	92.1–95.9
Female	93.5	90.1–95.8	94.3	92.1–96.0	96.3	94.4–97.5
Age group						
6 months–4 years	92.0	88.2–94.7	95.6	92.2–97.6	94.3	91.0–96.4
5–14 years	94.6	92.3–96.2	94.8	92.0–96.7	95.8	93.1–97.5
Correct adherence described by parent	93.7	90.0–96.1	98.5	96.7–99.3	96.5	94.1–97.9

The three principal reasons for non-participation in all three distributions were that the child was ill, that the child or household was absent, and that there was no parent/chaperone available to take the child to the distribution site. Some children were present at the site but refused to take the medication; this proportion rose from 4% of non-participating children during the first distribution to 14% during the third distribution. Correct adherence to the three-day course of DP was described in over 90% of recipients during each of the three distributions (Table 1).

### Height-based dosing

**Fig. 1:**
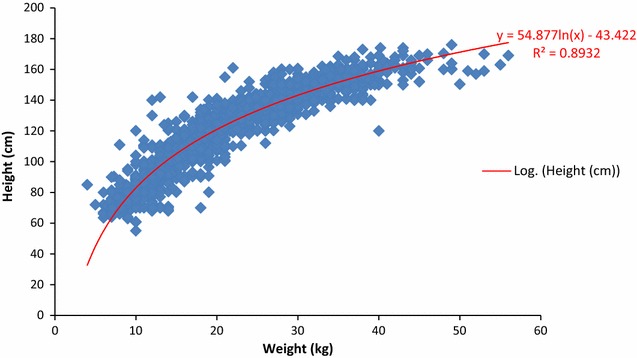
Weight and height of children, Ayilo camps, Uganda, 2015

Data were collected on 2268 participating children during the first IPTc distribution. Figure 1 shows these weight and height points, as well as the best-fit curve (R^2^ = 0.89). As Table 2 shows, this predictive model led to correct dosing in 79.3% of children, to under-dosing in 14.3% of children, and over-dosing in 6.4% of children.

**Table 2:** Dosing of dihydroartemisinin-piperaquine using a height-based prediction model, Ayilo camps, Uganda, 2015

	Correctly dosed		Under-dosed		Over-dosed	
	n	%	n	%	n	%
All children	1799	79.3	325	14.3	144	6.4
Gender						
Male	908	79.4	161	14.1	74	6.5
Female	891	79.2	164	14.6	70	6.2
Tribe						
Dinka	1447	79.6	245	13.5	125	6.9
Madi	303	78.3	70	18.1	14	3.6
Missing	49	76.6	10	15.6	5	7.8
Actual weight (kg)						
5–6	0	0	0	0	10	100
7–12	363	88.8	1	0.2	45	11.0
13–23	879	84.0	109	10.4	58	5.6
24–35	478	73.5	141	21.7	31	4.8
≥36	79	51.6	74	48.4	0	0

### Malaria prevalence

Among all age groups, the prevalence of parasitaemia increased by the final survey, approximately 2 months after the third and final IPTc distribution (Table 3). Children under the age of 5 years consistently had the lowest levels of parasitaemia, and parasitaemia was highest among children aged 5–14 years.

**Table 3:** Parasitaemia and gametocytaemia in community-based surveys, Ayilo camps, Uganda 2015

	Age in years											
	<5			5–14			≥15			Overall		
	N	%	95% CI	N	%	95% CI	N	%	95% CI	N	%	95% CI
Parasitaemia by microscopy												
March	257	5.1	3.0–8.5	253	8.7	5.8–12.9	278	6.1	3.9–9.7	788	6.6	4.9–8.8
May	232	4.3	2.3–7.8	246	10.2	7.0–14.6	258	6.6	4.2–10.4	736	7.1	5.3–9.3
July	266	6.0	3.7–9.6	263	13.7	10.0–18.4	271	10.7	7.5–15.0	800	10.1	8.1–12.6
September	267	15.1	12.1–18.7	274	26.7	20.9–33.6	269	18.7	13.7–25.0	810	18.7	16.0–21.7
Parasitaemia by microscopy or PCR												
March	257	11.3	7.9–15.8	253	15.0	11.1–20.0	278	12.6	9.2–17.0	788	12.9	10.6–15.8
May	232	11.2	7.7–16.0	246	15.5	11.4–20.5	258	10.9	7.6–15.3	736	12.5	10.0–16.0
July	266	13.2	9.6–17.8	263	18.6	14.4–23.8	271	17.3	13.3–22.3	800	16.4	13.8–19.3
September	267	19.1	14.8–24.3	274	31.0	25.8–36.8	269	25.7	20.8–31.2	810	25.3	22.1–28.8
Gametocytaemia												
March	257	2.3	1.1–5.1	253	1.6	0.6–4.2	278	1.1	0.4–3.3	788	1.7	0.9–2.9
May	232	0.4	0.001–3.0	246	0.4	0.001–2.8	258	0	–	736	0.3	0.001–1.1
July	266	0.8	0.2–3.0	263	1.5	0.6–4.0	271	1.9	0.8–4.4	800	1.4	0.8–2.5
September	267	2.1	1.1–4.0	274	2.7	1.1–6.3	269	1.7	0.5–5.0	810	2.2	1.3–3.5

In any given age group in any given survey, the rate of parasitaemia detectable by PCR that was not seen on microscopy ranged between 5 and 10%. For the most part, these cases represented low-level parasitaemia: the 90th percentile value of quantitative PCR for microscopy-negative, PCR-positive samples was 101 parasites/μl, and only 5% of microscopy-negative PCR-positive samples had quantitative levels over 200 parasites/μl.

**Fig. 2:**
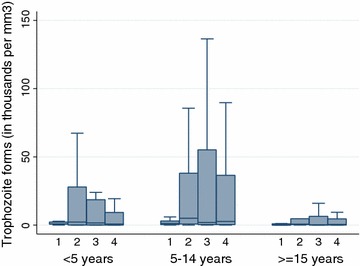
Parasite density by age group and by survey, Ayilo camps, Uganda, 2015

As expected, overall levels of parasitaemia were lowest among adults over the age of 15 (Fig. 2). On the other hand, the geometric mean parasite densities were higher for children aged 5–14 in three of the four surveys. When data from all four surveys were pooled together, geometric mean parasite density was 2261 parasites/µl (95% CI 1391–3674) among children aged 5–14 versus 847 parasites/µl (95% CI 436–1648) among children under 5 years (Wilcoxon rank-sum of log_10_-transformed densities p = 0.04).

**Fig. 3:**
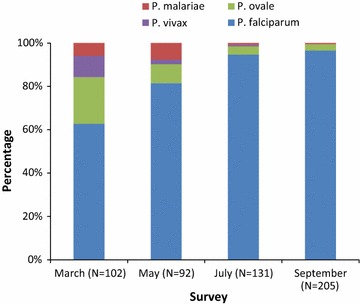
Distribution of *Plasmodium* species during community surveys, Ayilo camps, Uganda 2015

The distribution of *Plasmodium* species changed over the course of the programme (Fig. 3). Prior to the first IPTc distribution, non-falciparum species represented 37% of all cases. Prior to the second distribution, that proportion had fallen to 18%, and it was <5% thereafter.

### Safety

A total of 57 AEs were reported during the study period, including one serious adverse event (SAE). The sole SAE was a 12 year- old female who died unexpectedly at home 18 days after taking the third daily dose of DP during the first IPTc distribution. The death was noted in the community surveillance system. The information that follows was collected from her mother approximately 2 weeks after her death. She correctly took one dose of DP per day during 3 days of the first IPTc distribution. Approximately 10 days later, she developed fever and an itchy vesicular rash on her entire body, and after 2 days of these symptoms, she was seen in the health centre and diagnosed with varicella (an epidemic was ongoing in the camp at that time). She was prescribed zinc oxide cream, paracetamol and tepid sponging for symptomatic relief. She was seen in a follow-up visit 4 days later with no change in her rash; zinc oxide cream and paracetamol were continued. Two days later (18 days after receiving DP and 8 days after the beginning of her varicella), she developed swelling of her face and body and then experienced a sudden loss of consciousness at home. She went to the health centre and was immediately sent by ambulance to the hospital, where she was pronounced dead on arrival. Her mother reported that she had taken no other medications, including traditional medications. This SAE was judged as being possibly related to DP, but her concurrent illness with varicella provides an alternative cause to the SAE, and no definitive causal relationship could be established.

**Fig. 4:**
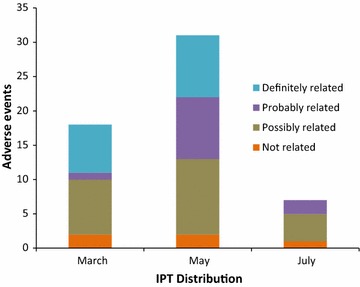
Causal relationship between dihydroartemisinin–piperaquine and adverse event following administration of dihydroartemisinin–piperaquine in the setting of intermittent preventative treatment, by distribution, Adjumani, Uganda, 2015

Among the AEs, 18 occurred after the first distribution, 31 after the second distribution, and seven after the third. All AEs reported were notified within 10 days of the given distribution. All AEs were judged to have either mild or moderate-intensity symptoms. After the first distribution, eight out of 18 reported AEs were judged as probably or definitely related to DP (Fig. 4). One case of musculoskeletal pain was judged as being probably related to DP. Among the AEs judged definitely related to DP, five were rashes, one was fever and one was vomiting. After the second distribution, nine of the 31 reported AEs were judged probably related to DP (one vomiting, one headache, one flu symptoms, three rash, one cough, one fever), and nine were judged as definitely related to DP (one urticaria, three rash, four vomiting, one itching). After the third distribution, two out of the seven reported AEs were judged as probably related to DP (one diarrhoea, one nausea). No AEs were judged as being definitely related to DP.

## Discussion

This is the first description of the successful implementation of an IPTc programme in a refugee camp. The reduction in the incidence of malaria among children in the target population was marked, especially in comparison to those persons not targeted by the programme, who saw their incidence of malaria increase significantly compared to the previous year.

The overall effect on parasite burden in the community was less impressive. Adults, who made up approximately 50% of the camp population, may have provided an ample parasite reservoir for continued transmission. Indeed, even with high coverage among the under-15 population as documented in the surveys, only 40–45% of the overall population of the camp was treated. Taken together, these data underscore the importance of expanding the target age groups for preventive interventions in similar contexts. Children aged 5–14 had considerable parasite burden in the prevalence surveys and a high incidence of clinical malaria. Mass Drug Administration (MDA) among all age groups would also have been a reasonable strategy in this context, and could be implemented in a future humanitarian emergency. Decreasing the interval between distributions (to 4 or 6 weeks) would also be a reasonable strategy, but would need to be weighed with the additional logistical and operational burdens in the setting of a humanitarian emergency.

In this context, a large increase in the prevalence of parasitaemia was seen among all age groups after the final IPTc distribution. This late-season peak occurred in the setting of particularly intense transmission across northern Uganda in the autumn of 2015, when multiple nearby districts declared malaria epidemics. This provides an example of the difficulty of planning such an intervention around a seasonal peak: a delay of one or two weeks in implementing the IPTc programme might have made a considerable difference at the end of the programme.

While specific details about the epidemiology of malaria in South Sudan are lacking, malaria remains a major problem there [14]. It is therefore possible to state with some certainty that the refugee population benefitting from this programme moved between two high malaria-endemic areas. The population in the Ayilo camps and the results of this study may not be generalizable to displaced populations moving from low-endemicity areas into high-endemicity areas.

The size of this intervention provides important programmatic and operational conclusions about similar programmes in terms of acceptability and safety. Programme coverage remained high throughout the distributions, and DP was well tolerated. The most important results of the intervention may well be about the safety profile of repeated doses of DP. Over 40,000 courses were administered (over 120,000 doses), and only one SAE was reported during the study period, a sudden death which is difficult to link directly to DP, particularly since it occurred in a child with an underlying medical condition after only one course. The overall pharmacovigilance system in the camp was strong during the intervention, and these detailed results are quite reassuring about DP’s safety profile.

Its long prophylactic period makes DP an obvious choice for such an intervention, but it does not come without difficulties. Largely because of concerns about under-dosing, recommendations have recently changed [15], and the practical aspects of administering five different weight-based doses in a mass campaign setting was cumbersome. To try to overcome this difficulty, a height-based dosing system for the drug was modeled, but the results were unsatisfactory, performing  particularly poorly for the smallest and the largest children.

These results come with certain limitations. The lack of historical data makes it difficult to interpret the overall impact of the intervention on malaria incidence. Specimen collection in the prevalence surveys was done in participants’ homes. While the quality of specimens appeared good, it is possible that some samples were sub-standard. All surveys were carried out using systematic sampling, which was at times challenging in the two camps because of the layout and orientation of homes.

## Conclusion

IPTc was successfully implemented in a refugee camp for the first time at a large scale. The knowledge gained should inform operational responses to crises of displaced persons both in Uganda and other high malaria transmission settings.

## Abbreviations

AE: adverse event
DP: dihydroartemisinin–piperaquine
IPTc: intermittent preventive treatment among children
IPTi: intermittent preventive treatment among infants
IPTp: intermittent preventive treatment among pregnant women
IRR: incidence rate ratio
LLIN: long-lasting insecticide-treated nets
MDA: mass drug administration
PCR: polymerase chain reaction
RDT: rapid diagnostic test
SAE: severe adverse event
SMC: seasonal malaria chemoprevention

## References

[1] WHO Global Malaria Programme (2012). WHO policy recommendation: seasonal Malaria Chemoprevention for *Plasmodium falciparum* control in highly seasonal transmission areas of the Sahel sub-region in Africa.

[2] WHO (2010). Policy recommendation on intermittent preventive treatment during infancy with sulphadoxine-pyrimethamine (SP-IPTi) for *Plasmodium falciparum* malaria control in Africa.

[3] World Health Organization (2012). Updated policy recommendation: intermittent preventive treatment of malaria in pregnancy using sulfadoxine-pyrimethamine (IPTp-SP).

[4] Nankabirwa JI, Wandera B, Amuge P, Kiwanuka N, Dorsey G, Rosenthal PJ (2014). Impact of intermittent preventive treatment with dihydroartemisinin-piperaquine on malaria in Ugandan schoolchildren: a randomized, placebo-controlled trial. Clin Infect Dis.

[5] Anderson J, Doocy S, Haskew C, Spiegel P, Moss WJ (2011). The burden of malaria in post-emergency refugee sites: a retrospective study. Confl Health.

[6] Charchuk R, Makelele KJP, Kasereka MC, Houston S, Hawkes MT (2016). Burden of malaria is higher among children in an internal displacement camp compared to a neighbouring village in the Democratic Republic of the Congo. Malar J.

[7] Lwin KM, Phyo AP, Tarning J, Hanpithakpong W, Ashley EA, Lee SJ (2012). Randomized, double-blind, placebo-controlled trial of monthly versus bimonthly dihydroartemisinin-piperaquine chemoprevention in adults at high risk of malaria. Antimicrob Agents Chemother.

[8] United Nations High Commissioner for Refugees. South Sudan situation-regional overview.

[9] Uganda Bureau of Statistics (2015). ICF International. Uganda malaria indicator survey 2014–2015.

[10] WHO (2010). Basic malaria microscopy-2nd edition learner’s guide.

[11] Allen EN, Chandler CI, Mandimika N, Pace C, Mehta U, Barnes KI (2013). Evaluating harm associated with anti-malarial drugs: a survey of methods used by clinical researchers to elicit, assess and record participant-reported adverse events and related data. Malar J.

[12] CIOMS (2005). Management of safety information from clinical trials.

[13] Mehta U, Allen E, Barnes KI (2010). Establishing pharmacovigilance programs in resource-limited settings: the example of treating malaria. Expert Rev Clin Pharmacol..

[14] Pasquale H, Jarvese M, Julla A, Doggale C, Sebit B, Lual MY (2013). Malaria control in South Sudan, 2006–2013: strategies, progress and challenges. Malar J..

[15] WorldWide Antimalarial Resistance Network (WWARN) DP Study Group (2013). The effect of dosing regimens on the antimalarial efficacy of dihydroartemisinin-piperaquine: a pooled analysis of individual patient data. PLoS Med.

